# ‘It’s like being conscripted, one volunteer is better than 10 pressed men’: A qualitative study into the views of people who plan to opt‐out of organ donation

**DOI:** 10.1111/bjhp.12406

**Published:** 2020-01-30

**Authors:** Jordan Miller, Sinéad Currie, Lesley M. McGregor, Ronan E. O’Carroll

**Affiliations:** ^1^ Division of Psychology Faculty of Natural Sciences University of Stirling UK

**Keywords:** organ donation, opt‐out consent, medical mistrust, bodily integrity, government control, thematic analysis

## Abstract

**Objectives**

To overcome the shortage of organ donors, Scotland and England are introducing an opt‐out organ donor registration system in 2020. This means individuals will be automatically considered to consent for donation unless they actively opt‐out of the register. Research has found that emotional barriers play a key role in donor decisions under opt‐in legislation, yet little is known about factors that influence donor decisions under opt‐out consent. Our objectives were to investigate attitudes towards organ donation and opt‐out consent from individuals who plan to opt‐out, and to explore the reasons *why* they plan to opt‐out.

**Design**

Qualitative interview study.

**Methods**

Semi‐structured interviews were conducted with 15 individuals from Scotland (*n* = 14) and England (*n* = 1) who self‐reported the intention to opt‐out of the register following the legislative change to opt‐out. The interviews were transcribed verbatim and analysed using thematic analysis.

**Results**

Three main themes were identified: (1) consent versus coercion, which describes the perception of freedom of choice under an opt‐in system and fears of ‘government interference’ and threatened autonomy under opt‐out, (2) self‐protection, encompassing fears of medical mistrust, bodily integrity concerns, and apprehension regarding the recipient selection process, and lastly, (3) ‘riddled with pitfalls’, which includes the notion that opt‐out consent may increase susceptibility of stigma and reproach when registering an opt‐out decision.

**Conclusions**

This study reinforces existing opt‐in literature surrounding medical mistrust and bodily integrity concerns. A threat to one’s autonomous choice and heightened reactance arising from perceptions of unwarranted government control have emerged as novel barriers.

**Statement of contribution**

***What is already known about this subject?***

- Although around 90% of individuals in the United Kingdom support organ donation, just 40% are actively registered as donors. As part of measures to improve rates of organ transplantation, Scotland and England are moving to an opt‐out organ donation consent system in 2020.
- Existing research has shown that feelings and emotions are important factors that influence donor relevant decisions under the current opt‐in system, but little research has explored potential deterrents under the new plans for opt‐out consent.
- Minimizing the number of people opting out of the donor register is key to ensure sustained rates of transplantation.

***What does this study add?***

- This study explored why people plan to opt‐out of the new system in Scotland and England.
- Medical mistrust and bodily integrity concerns remain as salient barriers under opt‐out laws.
- Fears of unwarranted government control and a perceived threat to one’s freedom of choice emerged as a novel barrier.

## Background

Though the number of registered organ donors continues to increase in the United Kingdom, there remains a serious shortage of donors to meet the demand for organ transplantation (NHSBT, 2019). As part of measures to increase transplant activity, many countries are advocating a policy change in the donor registration process from an opt‐in to an opt‐out system. Opt‐out legislation was introduced in Wales in 2015 and is now planned for implementation in Scotland and England in 2020. At present, Scotland and England operate under an opt‐in system; therefore, those willing to donate their organs can record their decision by joining the organ donor register. An opt‐out system eliminates the requirement for active registrations and follows deemed consent. This means that if an adult has not registered a donor decision (opt‐in or opt‐out), consent for organ donation will be deemed automatically. If an individual does not wish to be a donor, they may record this by opting out of the donor register.

Opt‐out consent legislation has now been in operation in Wales for over 4 years. The latest figures from 2018/2019 now show an increase in the number of donors and rates of transplantation (NHSBT, 2019). However, shortly post‐implementation, family consent for organ donation decreased and rates of family overrides increased. While a promising increase in family consent has recently been reported in Wales, this has also been observed in other UK countries that currently operate under an opt‐in system. To date, 6% of the Welsh population have opted out of the donor register. It is noteworthy, however, that a recent analysis of routine transplant figures from Wales found that 16.5% of potential donors had expressed the decision to opt‐out of organ donation (Noyes *et al.*, 2019). Only a small proportion of these individuals had actively registered their opt‐out decision, with the vast majority (76%) of individuals verbally expressing their opt‐out decision to family members. This may suggest that although recorded opt‐out registrations are low, the number of verbally expressed opt‐out decisions may be markedly higher.

It is interesting to note that the number of individuals’ registering not to donate their organs and opting out of the current opt‐in donor system in Scotland and England has substantially increased in the last 3 years (NHSBT, 2017, 2018, 2019). In total, 29,412 individuals had opted out of the donor register between 2016 and 2017. This increased to 456,262 opt‐out registrations between 2018 and 2019. This represents more than a 14‐fold increase in opt‐out registrations under the current opt‐in system. Minimizing the number of opt‐out respondents is critically important to maintain rates of transplantation; therefore, research that focuses on understanding why participants have opted out of the donor register is urgently required.

### Barriers to organ donation

A substantial body of international evidence has shown that feelings and emotional beliefs are crucial factors that influence donor relevant decisions under opt‐in legislation (Morgan, Stephenson, Harrison, Afifi, & Long, 2008; O'Carroll, Foster, McGeechan, Sandford, & Ferguson, 2011; Shepherd & O’Carroll, 2014). Although the aforementioned studies used quantifiable measures of emotions, qualitative literature has reinforced these findings (Irving *et al.*, 2011; Newton, 2011). In particular, participants reported fears that donation would cause physical harm, and jeopardize the integrity of their body. Distrust in the health care system and fears that donors would receive substandard care also emerged as important deterrents. Given the relatively novel nature of opt‐out consent legislation in the United Kingdom, few studies have investigated the factors deterring donors under these laws. Although recent research has shown that emotional barriers are significantly heightened for individuals who signal an intention to opt‐out, this study utilized a quantitative measure of emotional barriers, which may limit the depth of understanding into these complex emotive factors (Miller, Currie, & O'Carroll, 2019). Ultimately, obtaining a rich and nuanced understanding of these factors using qualitative methodology, from a prospective point of view, may enable researchers to identify modifiable barriers that could be targeted before the introduction of opt‐out consent. This has the potential to reduce the number of opt‐out registrations. This is particularly important, as recent research from The Scottish Parliament found 22% of individuals plan to opt‐out of the new donor system (Scottish Parliament, 2018). Notably, this figure is higher than baseline opt‐out intentions reported in Wales during pre‐implementation assessments (Welsh Government, 2012).

The present study had two aims: (1) to examine attitudes towards the current opt‐in system and the planned opt‐out system from individuals who plan to opt‐out and (2) to gain an in‐depth understanding of why people plan to opt‐out of the donor register.

## Method

### Design

This study involved one‐to‐one, semi‐structured telephone interviews. Telephone interviews were primarily selected due to the potentially diverse geographic location of interviewees; as such, this was a cost‐effective and timely method of interviewing individuals across Scotland and England. Moreover, evidence suggests that telephone interviews are effective mediums when exploring potentially sensitive topics (Block & Erskine, 2012). Guidelines on sample sizes in qualitative research were applied to inform recruitment. As the study aims are relatively narrow and concern the views of a specific, small sample of individuals who plan to opt‐out of the organ donor register, recruitment of approximately 15 participants was considered to provide sufficient ‘information power’ to obtain new knowledge regarding attitudes towards opt‐in and opt‐out consent legislation (Malterud, Siersma, & Guassora, 2016).

### Eligibility and recruitment

**Figure 1:**
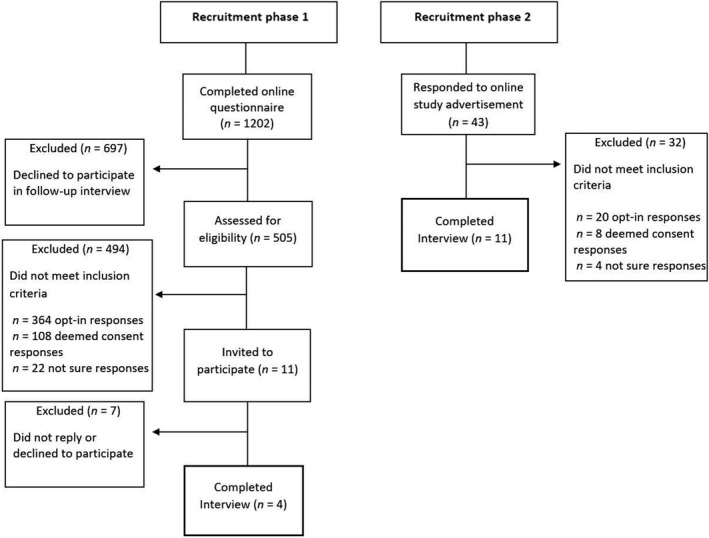
Recruitment flow diagram.

Individuals aged over 18 years, who live in Scotland or England, and who plan to opt‐out of the organ donor register following the introduction of deemed consent legislation were eligible to participate. Recruitment for this study occurred in two phases (see Figure 1).

Phase one involved recruitment of participants who had (1) completed a questionnaire about organ donation (see Miller *et al.*, 2007), (2) indicated they would *opt‐out* of the donor register if laws change to an opt‐out system, and (3) gave consent to participate in a follow‐up interview study. Approximately 6 months later, these participants (*n = *11) were sent an email with information on the study and a URL link to a Qualtrics survey (https://www.qualtrics.com/uk/). This was used to present the study information, to collect informed consent and basic demographic information (age, gender, and country of residence). Participants were also asked to select a suitable date and time and to provide a contact telephone number. Of the (*n* = 11) participants who were invited to participate, four completed the interview, and the remaining seven did not reply or declined to participate.

Phase two involved opportunistic sampling via advertisements presented on the University of Stirling Portal page, and the social media websites, Facebook, and Twitter. The advertisement presented information on the study and a link to the same Qualtrics survey used in Phase 1 to obtain informed consent and arrange the interview. To ensure that only people who plan to *opt‐out* of the donor register were recruited, a measure of anticipated donor status was obtained. As part of the Qualtrics survey, participants were presented with details on the planned opt‐out system (available as Supplementary Information) and were asked, ‘If the organ donation laws in your country change to an opt‐out system, what would your choice be?’ Participants were presented with the following response choices: (1) I would opt‐in (I want to be an organ donor), (2) I have no objection to donating my organs (deemed consent to be an organ donor), (3) I would opt‐out (I do not want to be an organ donor) and (4) not sure. In total, a further 11 opt‐out respondents were recruited and completed the interview. As an incentive, all participants were offered a £5 Amazon voucher.

### Participants

Fifteen individuals who self‐reported the intention to opt‐out of the organ donor register participated in this study. Of the 15 participants, nine (60%) were female and six (40%) were male. The mean age of participants was 45.13 (*SD* = 19.43, range 18–83), 14 participants were resident in Scotland, and one participant was resident in England. Demographic information is provided in Table 1.

**Table 1:** Participants’ demographic characteristics

Name	Age (years)	Gender	Resident	Current donor status
Anna	49	Female	Scotland	Registered donor
Emily	45	Female	Scotland	Not registered
Olivia	83	Female	Scotland	Not registered
Victoria	60	Female	Scotland	Not registered
Andrew	19	Male	Scotland	Not registered
Robert	41	Male	Scotland	Not registered
Madison	54	Female	Scotland	Not registered
Lauren	42	Female	Scotland	Not registered^a^
Robyn	20	Female	Scotland	Not registered
Erin	33	Female	Scotland	Not registered
Charlotte	28	Female	Scotland	Not registered
James	57	Male	Scotland	Not registered
Luke	22	Male	England	Opted out^b^
Mason	46	Male	Scotland	Not registered
William	78	Male	Scotland	Not registered

Note

Participants names have been replaced with a pseudonym.

a Participant was a registered donor in Australia but was not registered in the United Kingdom

b Participant had recorded the decision not to donate their organs. This option was made available under the opt‐in donor system in late 2015.

### Procedure

Ethical approval for this study was provided by the University of Stirling Ethics Panel. The interviews were conducted via the telephone between August 2018 and February 2019 by the author (JM) and lasted on average, 32 min (range = 18–46 min). A semi‐structured interview guide was used flexibly throughout the interviews (available as Supplementary Information). The interview schedule encompassed open questions regarding participants’ attitudes towards the current opt‐in and planned opt‐out donor system. In recognition of the potential sensitivity of the topic, the researcher initially explained the purpose of the study and affirmed there to be no right or wrong answers to any of the questions being asked. When exploring participants’ views towards the forthcoming changes to organ donation legislation, a clear verbal definition of the present opt‐in donor system and the planned opt‐out system was provided to each participant. The interview initially commenced with a broad, non‐directive question which inquired about participants’ personal views on organ donation. The core questions within the topic guide were designed to function as opening questions to facilitate a fluid interview, and to promote the exploration of individual factors of importance. Throughout, prompts and follow‐up questions were used to elaborate on salient responses. Before recruitment commenced, pre‐testing of the interview schedule was conducted between members of the research team in a pilot interview.

A number of recommended techniques for effective telephone‐based data collection were applied throughout the interviews (Drabble, Trocki, Salcedo, Walker, & Korcha, 2016). This involved expressing regard for participants’ contributions and providing non‐judgemental affirmations when participants shared sensitive viewpoints. In addition, time orientating statements were used to promote continued engagement towards the end of the interview ‘We’re just about finished so thanks for your patience, I’ve just got a few more questions left’. At points during the interview, participants’ responses were summarized to enhance accuracy and to enable the elaboration of potentially ambiguous points of discussion. At the end of the interview, participants were thanked for their contribution and verbally debriefed. An electronic copy of the debrief form and a £5 Amazon voucher was then sent to participants email addresses.

### Data management and analysis

The data were analysed using a thematic analysis, as described by Braun and Clarke (2006). Throughout, a largely essentialist/realist approach was adopted, which communicates experiences, language, and meaning from the participants perspective (Braun & Clarke, 2006). The interviews were audio‐recorded, transcribed verbatim by the author (JM), and anonymized by allocating each participant a pseudonym. During the transcription process, each interview was listened to on multiple occasions and quality checked through repeated reading to facilitate immersion (Bird, 2005). Three of the transcripts were reviewed for accuracy by another researcher. The qualitative software Quirkos https://www.quirkos.com/index.html was used to manage the data. During the coding process, interesting features throughout the data were highlighted and assigned a preliminary code or ‘quirk’. These preliminary codes were then reviewed and organized into respective themes and sub‐themes by the first author (JM). The themes were identified in accordance with their salience and prevalence to the research questions using an inductive, data‐driven approach. In acknowledgement of the primary author's existing knowledge of the organ donation literature and the influence this may have on the interpretation of the data, a second researcher (SC) reviewed the themes and sub‐themes to ensure these were represented within the data and to enhance the ‘trustworthiness’ of the analytic process (Shenton, 2004). The resulting themes and illustrative excerpts were then presented and discussed openly with all members of the research team to ensure there was sufficient evidence to substantiate each theme. Further refinements to themes were made during this process until a consensus was reached.

### Findings

**Figure 2:**
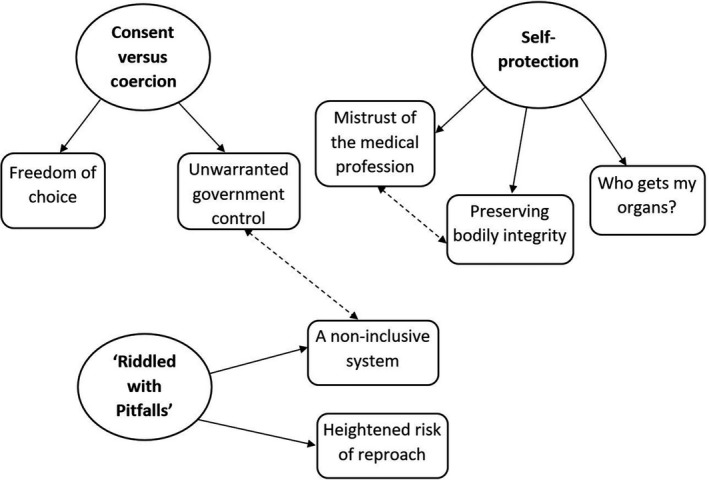
Thematic diagram of key themes and sub‐themes. Single directional arrows represent the respective sub‐themes; bi‐directional dotted arrows indicate a relationship between themes.

Three overarching themes were identified within the transcripts: (1) consent versus coercion, (2) self‐protection, and (3) ‘riddled with pitfalls’. A thematic diagram of the themes and respective sub‐themes is presented in Figure 2.

#### Theme one: Consent versus coercion

This theme encompasses participants’ attitudes towards the changing representation of choice and consent between the current donor system and the future opt‐out system. The non‐intrusive nature and freedom of choice offered within the opt‐in system were considerably favoured (sub‐theme 1.1). In contrast, the planned opt‐out system, where consent is deemed automatically in the absence of a recorded decision, was perceived as forceful and intrusive (sub‐theme 1.2).

##### Freedom of choice (sub‐theme 1.1)

The current opt‐in registration system was viewed as facilitating freedom of choice regarding the decision to register as an organ donor. This decision was described by Andrew as one of great importance ‘you can’t make that decision lightly’. As such, actively seeking out the means to register demonstrates consent to have been informed and the decision, a voluntary choice. Throughout participants’ narratives, freedom of choice was conceptualized as being one’s lawful right. This was juxtaposed with the proposals for opt‐out consent, considered as invasive and a threat to individual responsibility. This is highlighted by Luke, who describes his experience as a nurse to emphasize the importance of informed consent:

As a nurse before I do anything I ask for consent so I don’t just like go and take somebody’s blood and then go ‘is that okay that I’ve just taken your blood?’ so in my opinion, you need to ask for consent and that’s what it [an opt‐in system] does, it asks for consent. (Luke)

Moreover, registering under an opt‐in system was reported to act as irrefutable confirmation of one’s donor wishes. As such, participants felt this may reduce uncertainty and distress when next of kin are confronted with the emotive decision to donate their loved one’s organs.

I mean an opt‐in I guess then you’ve definitely got people saying I’m happy for you to take my organs and maybe that makes it easier for people or parents or people in the position where they’re unsure of that person, that persons’ wishes so maybe it makes it easier as part of a grieving process if somebody’s made that decision to give their organs. (Madison)

##### Unwarranted government control (sub‐theme 1.2)

For some participants, the opt‐out system was viewed as forceful and as Victoria states ‘like being conscripted’ into organ donation. For many, this signified unwarranted government interference into a highly personal decision. As such, the opt‐out system was perceived to give the government illegitimate control and ownership over an individual’s body after death. Anna expands on her concerns below:

I would feel like erm because I said earlier that erm my body was y’know it was like presumed part of the state rather than my own, because if I don’t have that written down somewhere then that can be taken away from me, my opinion my decision can be taken away from me by the state and overruled by the state. (Anna)

Many participants also felt that the opt‐out system will force them to take action [register an opt‐out decision] to safeguard their body from donation. While some participants acknowledged the choice to opt‐out of organ donation, implicit throughout the data was a sense of injustice that such protective action was now necessary.

Even though there is y’know that erm… you can go and make the decision to get your name taken off the register it’s still well why should I have to go and take my name off a register? I don’t want to do this why are you saying that I do? Don’t make a decision for me. (Victoria)

Some participants viewed the basic principles of consent to be disregarded by the opt‐out system. Consent was epitomized as something informed and unambiguous. As Robyn states ‘assumed consent can’t really be considered consent’. Andrew expands on this below and describes why a system that automatically deems consent for donation is concerning:

I mean it comes down to those two words doesn’t it? at the end of the day presumed consent what a slippery slope that is, because y’know presumed consent you could absolutely never get away with presumed consent in damn near every other area of life there’s not a chance you could go to court and say ‘actually well y’know I had presumed consent’ it doesn’t work like that. (Andrew)

These comments seem to suggest that deemed consent is viewed by some participants as an oxymoron. The reference to a court of law highlights the magnitude of informed consent in society. In turn, presuming consent for something as important as organ donation was deemed unlawful. Reflecting on the sensitive nature of consent, Robert felt it was inappropriate ‘for the government to overrule ethics’ and presume that individuals who have not opted out automatically consent to organ donation. Below, Andrew uses an example of consent in society to highlight its delicate nature:

If you take what it comes down to judicial reviews of things like consent when it comes to sexual interaction, consent is massive within the terms of sexual language, it is because consent is more in the mind of somebody explicitly saying yes and if they do not say yes then it is not considered consent, you must physically say yes. (Andrew)

#### Theme two: Self‐protection

Participants expressed a number of fears around organ donation that played an influential role in their donor decision. These were predominantly expressed around the overarching notion of protection, which manifested into three distinct sub‐themes concerning the protection of one’s *life*, *body*, and *organs*. This encompassed: mistrust of the medical profession (sub‐theme 2.1) which symbolized fears over protection of life, preserving bodily integrity (sub‐theme 2.2) which represented the importance of protecting the body during and after the time of death, and finally, concerns of the organ allocation process (sub‐theme 2.3) which represented the desire to protect one's organs from potential misuse.

##### Mistrust of the medical profession (sub‐theme 2.1)

Negative attitudes and suspicions towards the health care system and medical staff were an important factor in the decision to opt‐out. Throughout, participants voiced reservations about the quality of care provided by medical professionals in the event of life‐threatening illness or injury. As such, a sense of uncertainty regarding life‐saving decisions was implicit within participants’ narratives. The following extract from Emily highlights her fears that doctors may place greater value on procuring organs rather than saving an individual’s life:

I have the fear that if somebody needs an organ and somebody’s sitting there you know kinda in deaths door and somebody else needs an organ then they might make a call that well y’know rather than save this 45 year olds life then we could let this person just go gently and this young 18 year old who’s desperate for a heart here could get it. (Emily)

There were also concerns that doctors may initiate the process of organ retrieval prematurely. As a result, some participants expressed fears that they would be alive while their organs were being removed.

If you were in hospital and they think you’re dead but you’re not and they start whipping parts out, that’s a fear whether it’s rational or not I don’t know. (James)

At times, there appeared to be a conflict between participants’ emotional and rational evaluations of these beliefs, as demonstrated by Madison:

I guess it’s the ‘what ifs’, it’s the y’know what if you aren’t really dead and all this sort of nonsense and the sensible side of me is telling me not to be stupid but the not so sensible side y’know is still questioning it… (Madison)

##### Preserving bodily integrity (sub‐theme 2.2)

This sub‐theme represents the belief that the integrity of the body is irreparably jeopardized as a consequence of organ donation. Throughout participants’ narratives, the desire to remain whole in life and in death appeared to be an influential factor in the decision to opt‐out. Victoria, for example, felt that if her organs were donated, her body would no longer be whole, and the finality of her death would be endangered;

When I die I want all of me to die, not a bit of me living on here, I think erm it’s not like erm… it sort of feels like as if you wouldn’t be properly dead do y’know what I mean and then you think well…I want all of me, I want to leave the world the way I came with all the bits that I came with. (Victoria)

Participants also expressed worries over the envisioned brutality of organ donation and described fears that their body would remain in a damaged and disfigured state. As such, many participants expressed a desire to protect their body from further harm after death. These fears appeared to be compounded by the belief that as the donor is no longer alive, doctors may not display the necessary respect to the body after death. In the following extract, Anna compares organ donation to a surgical procedure to highlight her fears:

I mean it’s not going to be like surgery if you’re going in for surgery, they’re not going to take their time to go in and mend an organ or mend a part of your body they’re going to go in for the organ they need to then save someone’s life. So erm for me I would be scared they just went in an(d) made a mess of my dead body to take the organ that they needed without having any respect for me. (Anna)

For others, the preservation of bodily integrity was both personally important and represented a value shared among family members. This manifested for some participants, into feelings of unease at the thought of their loved one’s body being used for donation and the repercussions of this decision on their grieving process. Below, Charlotte explains that following her father’s sudden death, knowing that his body remained intact was comforting;

I think being able to go to somewhere, where I know that he is there and that he is whole and I can speak to him erm it really just like puts my mind at ease and it’s just quite nice […] he is there in his entirety and that’s really important to me. (Charlotte)

##### Who gets my organs? (sub‐theme 2.3)

Participants also reported misgivings about the organ allocation process as an influential factor in their decision. Many expressed a desire for their organs to be gifted to someone who would make a positive contribution to their life and the wider community. The absence of control over this process led to apprehension that one’s organs would be allocated to a recipient who was ultimately undeserving of such a precious gift. James expands on this view below:

I would want to know that the people receiving the organs were deserved and no self‐abusers i.e. alcoholics erm I don’t want to tell anybody else how to run their life but if they are going to be given the gift of an organ by somebody they have to accept it with some humility and look after themselves. (James)

#### Theme three: ‘Riddled with pitfalls’

Specific concerns regarding the implementation, management, and inclusivity of the opt‐out system were an important feature within participants’ narratives. Two prominent concerns were identified: heightened risk of reproach when registering to opt‐out (sub‐theme 3.1) and a non‐inclusive system (sub‐theme 3.2).

##### Heightened risk of reproach (sub‐theme 3.1)

Many participants described stigma associated with the decision *not* to be an organ donor. Charlotte, for example, recounted personal experiences of judgement from family or friends ‘four people said I was like y’know a bit of a mean person’. As the majority of people are seen to be supportive of organ donation, under the new system, the act of recording an opt‐out decision was anticipated to increase the likelihood of harmful judgements and ridicule from other people.

If you’re going in to opt‐out of something that traditionally people don’t really opt‐out of you’re opening yourself up to a lot of judgement and a lot of uhm just remarks from possibly the people who are part of the kind of system[…]people don’t necessarily want all of their dirty laundry aired out in public and it’s seen as quite a taboo thing at least in my generation to not want to donate your organs. (Robyn)

Considering concerns of negative appraisals, the introduction of deemed consent was perceived to make registering or voicing an opt‐out decision significantly more challenging. For example, Victoria felt that ‘people are being coerced into being organ donors’ and they may ‘feel afraid to say that’s not what they want’. Other participants were worried about heightened pressure when making donor decisions for next of kin. Below, Erin explains her worries about making a donor decision on her husband’s behalf following the introduction of opt‐out consent:

To have to y’know stand against all the doctors and all the nurses because the image that we always get is that they’re always for it and y’know morally in the media it’s something that you should do because it’s the right thing to do, so to then stand up and say ‘no I disagree I don’t want it to happen’ and y’know everyone’s waiting and lives are y’know on the brink and you’ve decided no when it’s always been assumed because he didn’t opt‐out. I think that would be a really hard decision to make y’know in that situation that’s when things really fall apart and people don’t recover from that kind of thing. (Erin)

##### A non‐inclusive system (sub‐theme 3.2)

Participants also criticized the inclusivity of the opt‐out system, in particular for vulnerable groups, namely those with poor health literacy, older adults, immigrants without a comprehensive command of the language, and individuals with limited capacity to comprehend the implications of the new system. As such, concerns were raised that individuals ‘that don’t actually have a voice for themselves’ would be automatically registered as organ donors against their wishes.

To opt‐out that requires action, many many people are really inactive it’s the road to hell is littered with good intentions and whilst there would be many people and let’s be blunt about it there are people who are maybe not as well read or maybe not as erudite as they possibly could be who will have been deceived by this, there’s also many many people who may be unable to make a really conscious considered decision (William)

This was further compounded by the envisaged practical challenges to registering an opt‐out decision. As Olivia states ‘it’s easier to sign‐up than to sign‐out of something’*.* Consequently*,* participants expressed worries that an online system would be challenging to operate and that it would be purposely difficult to opt‐out.

Where’s the system to go an(d) opt‐out, is it easy to navigate? if it’s like any of the other government based websites it’s horrendous erm they’ll have no call centres because it will cost you one pound fifty a minute and people will think ‘oh heck I’m not paying that to go an talk to somebody’. They will make it as awkward as possible to opt‐out in my opinion. (James)

## Discussion

This novel research contributes to the existing literature by investigating attitudes towards the existing opt‐in system and the planned opt‐out donor system from the unique perspective of individuals who plan to opt‐out of organ donation. The findings highlight the importance of autonomy and individual responsibility over one’s donor decision and suggest this to be threatened under opt‐out consent. The study also offers important insights into factors that may influence the decision to opt‐out of organ donation. Notably, perceptions of government control and emotional factors around mistrust of medical professionals, preservation of bodily integrity, and worries regarding the allocation of donor organs appear to play a considerable role.

### Consent versus coercion

Under the current opt‐in system in Scotland and England, consent for donation is recorded following an individual’s decision to sign‐up and join the donor register. As this requires one to ‘actively seek out the means’ to register, this was reported to signify that consent was a considered and conscientious decision. This was important for two main reasons; primarily, it enables participants to exercise their autonomy regarding the decision *not* to register as a donor. This is because under the opt‐in system ‘no presumptions’ are made regarding the absence of an active donor decision. However, under opt‐out consent, the absence of an active donor decision [opt‐in or opt‐out] will now be used to indicate consent for donation via deemed consent. Secondly, actively registering as a donor under the current opt‐in system was considered to provide explicit and unambiguous evidence of one’s donor intentions. This was believed to reduce uncertainty when family members are approached regarding donation. As the donor register represents clear evidence of one’s intentions, participants felt this may, in turn, make it ‘easier’ for grieving family members to proceed with organ donation. This finding is consistent with recent consent figures from countries with opt‐in laws, which reports that families are considerably more likely to agree to donation if their loved one had registered as a donor. However, in instances where no decision has been recorded, a 42% increase in family or next‐of‐kin refusal is observed and consent for organ donation is authorized in just over 50% of such cases (NHSBT, 2019).

Family refusal rates for donation are a central factor that limits the potential for UK organ transplantation (NHSBT, 2016). This remains a considerable issue under opt‐out laws. In a recent study which analysed transplant data from Wales, a number of notable findings regarding family consent under opt‐out laws were reported (Noyes *et al.*, 2019). The findings revealed that when an opt‐in decision was registered, 16.4% of families overrode their loved ones recorded decision and refused consent for donation. However, in instances where deemed consent was applied due to the absence of an opt‐in or opt‐out decision, family overrides more than doubled to 39.1%. In sum, this confirms that the family has a critical influence on consent and rates of donation in both, instances where a recorded donor decision has been made and under deemed consent. As such, a timely investigation of the factors influencing family consent is crucial.

Though participants favoured the opt‐in system due to its non‐invasive nature, when talking about the proposed opt‐out system, the word choice of ‘conscripted’ and ‘enforced’ suggests that participants view an opt‐out system as a forceful method of obtaining consent for organ donation. As consent will soon be deemed automatically for those who have not registered a donor decision, some believed this would ‘remove their choice and their voice’. The concept of autonomous choice fundamentally concerns the right for an individual to exercise control over their lives and decisions (Deci & Ryan, 1987). A principal component of autonomy is the provision of informed consent and the capacity for an individual to make choices and take action without coercion from external factors (Rendtorff, 2008). This may explain why those who plan to opt‐out view the government as a coercive, external factor that constrains their autonomous choice.

Moreover, this may be associated with the concept of reactance, an unpleasant motivational response that arises following a perceived threat to one’s freedom (Brehm & Brehm, 1981). In response, individuals are driven to take action to protect the notion they perceive as under threat (Rosenberg & Siegel, 2018). As such, people who perceive the opt‐out system to threaten their freedom of choice may be driven to opt‐out to preserve their autonomy. Perceptions of reactance can be exacerbated by language that is perceived as being particularly controlling and forceful (Miller, Lane, Deatrick, Young, & Potts, 2007). Within the current study, it is interesting that participants made frequent reference to the word ‘presumed’ and appeared vexed at the idea of consent for organ donation being nonchalantly presumed by default. This was particularly apparent during Andrew’s example of consent for sexual interaction; ‘there’s not a chance you could go to court and say actually well y’know I had presumed consent’*.* This suggests that participants view the notion of deemed consent as paradoxical and incompatible with the delicate nature of consent. As such, cautious use of language may be required when promoting opt‐out consent.

### Self‐protection

Within the current study, emotional barriers associated with the preservation of one’s life, body, and organs were influential factors in the planned decision to opt‐out. Throughout, unease regarding the medical and health care system was predominantly associated with the notion that doctors may hasten death to procure organs for those on the waiting list. Such comments illustrate that recipients on the waiting list are viewed more favourably than potential donors in the event of life‐threatening injuries. The findings also suggest unease regarding the organ allocation process. Often, participants conveyed fears that they had no control over the allocation of donor organs and could not guarantee their organs would be donated to individuals who ‘deserved’ such a gift. Similar factors have emerged in existing qualitative studies as key deterrents for individuals registering as a donor under an opt‐in system (Irving *et al.*, 2011; Newton, 2011). Notably, some participants in this study attributed these fears to depictions of organ donation in films and television programmes. Previous literature supports this finding and suggests that barriers and myths towards donation may be fuelled by sensationalist misrepresentations of organ donation in the media (Morgan *et al.*, 2005). Given the alarming rate at which misinformation is now disseminated, careful consideration of future organ donation depictions should be encouraged (Lewandowsky, Ecker, Seifert, Schwarz, & Cook, 2012).

In recognition of this, NHS Blood and Transplant (NHSBT) currently provides a ‘myth‐busting’ feature on their webpage as a method of dispelling myths and correcting misinformation surrounding organ donation: https://www.organdonation.nhs.uk/helping-you-to-decide/about-organ-donation/myths-about-organ-donation/. Recent work has examined the impact of this campaign on self‐reported organ donor intentions (Miller *et al.*, 2019). The study found that dispelling harmful organ donation myths increased intentions for those with favourable attitudes towards organ donation, namely participants who plan to actively opt‐in to the register and those who plan to follow deemed consent when opt‐out legislation is introduced. However, for those who plan to opt‐out, the myth‐busting intervention decreased intentions to donate. Notably, individuals who plan to opt‐out also exhibited heightened negative emotional barriers towards organ donation. The authors concluded that dispelling myths using corrective factual information may have unintentionally acted to prime individuals pre‐existing negative beliefs and fears around organ donation.

At present, the most effective components of organ donation campaigns remain unclear, though emergent evidence suggests that emotive campaigns may be more effective (Feeley & Moon, 2009; Rodrigue, Fleishman, Vishnevsky, Fitzpatrick, & Boger, 2014). Given the powerful role of feelings and emotions in relation to organ donor decisions, future research evaluating such campaigns would be beneficial.

The preservation of bodily integrity after death was also a crucial factor driving the decision to opt‐out of organ donation. Participants described fears that proceeding with donation would ‘make a mess’ of their dead body. Throughout, word choices of ‘mutilated’ and ‘tampered’ suggest participants view donation to cause unwarranted physical harm to their body. As such, this led to fears that the body would be left damaged and piecemeal during the afterlife, while for others, it signified death to be somewhat incomplete. Interestingly, although often attributed to religious beliefs, with the exception of one participant (Anna), all interviewees in this study stated that they held no religious beliefs.

A core principle of bodily integrity is the notion that one’s body signifies an ‘untouchable core’ (Rendtorff, 2008). Adopting a bioethics perspective, a fundamental factor in the maintenance of these values is the provision of autonomy and informed consent. Importantly, these factors are also crucial for the preservation of harmonious relationships between individuals and health care professionals (Delgado, 2019). Given that bodily autonomy and informed consent were perceived as being threatened under an opt‐out system, concerns regarding bodily integrity and medical mistrust may be exacerbated following the enactment of new donor laws.

In sum, although these factors have emerged as pivotal deterrents towards donor relevant decisions for nations with opt‐in donation systems (Morgan *et al.*, 2008; O'Carroll *et al.*, 2011; Shepherd & O’Carroll, 2014), the current study suggests that emotional barriers are also important factors for people who intend to opt‐out of organ donation.

### ‘Riddled with pitfalls’

A number of key concerns regarding the implementation and management of the opt‐out system arose. In particular, the act of registering an opt‐out decision was envisaged to heighten vulnerability to reproach. Participants described occasions in which they had experienced judgement and stigma from friends and family regarding their donor decision. These negative experiences may have perpetuated the anticipation of reproach when communicating a donor decision under opt‐out consent. Our findings are similar to that of Breitkopf (2006), in which anticipated negative experiences decreased the intention and willingness of individuals to discuss their donor decisions with family. Although this study measured communication of donor wishes under an opt‐in system, it highlights the importance of perceived negative expectations during face‐to‐face discussions of one’s donor decision. Under an opt‐in system, the decision *not* to be an organ donor was regarded by participants in this study as one ‘you can kinda avoid’. As individuals will soon have to take action to opt‐out of the organ donor register, further examination of these factors is required.

### Limitations

There are limitations to this study that should be acknowledged. Although we aimed to recruit participants from both Scotland and England, our sample consisted almost exclusively of individuals living in Scotland and only one interviewee from England. Therefore, our findings may largely reflect the experiences of individuals living in Scotland. However, there were a number of shared themes between our interviewee from England (Luke) and our cohort from Scotland, namely the importance of informed consent and concerns over violations of bodily integrity. In future, a more geographically representative sample would enable the exploration of factors that may be unique to Scottish and English residents. Moreover, participants’ ethnicity was not explicitly recorded; therefore, inferences regarding cultural variations in attitudes towards organ donation and opt‐out consent policies cannot be made. Existing research has found specific barriers in relation to maintaining bodily integrity after death as an important deterrent to organ donation for individuals of different ethnic and faith groups (Morgan *et al.*, 2013). As such, future research that explores attitudes towards opt‐out consent with a more diverse sample of individuals from multi‐faith and multi‐ethnic groups is required. A potential limitation also pertains to the use of telephone interviews. Although selected due to the potential widespread geographic location of participants, telephone interviews are criticized due to the absence of visual and non‐verbal cues (Novick, 2008). This predominantly concerns the loss of non‐verbal data including gestures and facial expressions which can incur challenges in establishing rapport and may limit the depth of responding. To minimize these potential limitations, the interviewer employed various techniques including active listening, expressing appreciation of participants’ dialogue through non‐judgemental affirmations, and time orienting statements. Collectively, use of these approaches has been found to facilitate the development of trust and rapport between participants (Drabble *et al.*, 2016; Weger, Castle Bell, Minei, & Robinson, 2014).

### Implications and future directions

This study has a number of potential implications for policymakers and health care professionals in Scotland and England. Our findings emphasize the importance of a clear and active decision in reducing family uncertainty and refusal for donation. As family refusal remains a significant problem under opt‐out legislation, future studies investigating this are required. A perceived threat to one’s autonomy and freedom of choice have emerged as a key barrier under opt‐out consent. The development and evaluation of targeted campaigns to reduce these concerns are important. Specifically, given its role in perceptions of reactance, evaluation of the word ‘presumed’ may be a useful next step. Lastly, to reduce fears of reproach and reduced inclusivity, it is essential that individuals planning to opt‐out are able to register that choice in a discreet, simple, and efficient manner. In light of these concerns, when promoting opt‐out consent in Scotland and England, clear guidance on the procedure for registering an opt‐out decision is required.

### Conclusion

Our findings confirm that as in the existing opt‐in organ donation literature, medical mistrust and concerns over preserving bodily integrity are also important barriers under the proposed opt‐out legislation. Barriers specific to opt‐out legislation include heightened government control, loss of autonomy, and fears of stigma when registering to opt‐out. Attempts to better understand and address these barriers before the introduction of opt‐out consent are vital.

## Conflicts of interest

All authors declare no conflict of interest.

## Author contribution

Jordan Miller, MSc (Conceptualization, Data curation, Formal analysis, Investigation, Methodology, Project administration, Writing – original draft, Writing – review & editing); Sinéad Currie and Ronan E. O'Carroll (Conceptualization, Methodology, Supervision, Validation, Writing – review & editing) and Lesley M McGregor (Formal analysis, Supervision, Validation, Writing – review & editing).

## Supporting information


**Supporting information 1**: Information presented to participants regarding proposed changes to organ donor laws.

**Supporting information 2**: Semi structured interview schedule.

## Data Availability

The anonymized data that supports the findings of this study are available from the corresponding author upon reasonable request.
