# Supplementary material for: ‘It’s like being conscripted, one volunteer is better than 10 pressed men’: A qualitative study into the views of people who plan to opt‐out of organ donation
Source: Br J Health Psychol. 2020 Jan 30;25(2):257–74. doi: 10.1111/bjhp.12406 (PMC7216962; doi:10.1111/bjhp.12406)
Supplement: Supplementary file 1 — Supporting information 1: Information presented to participants regarding proposed changes to organ donor laws. [file BJHP-25-257-s001.docx]

**Supporting information 1: Information presented to participants regarding proposed changes to organ donor laws.**

The Scottish and English Governments are planning to change the process of registering as an organ donor. Currently, anyone in Scotland, England and Northern Ireland wishing to donate their organs in the event of their death must ***opt-in*** and join the organ donor register.

Scotland and England may change to an ***opt-out*** system of organ and tissue donation. This means that if you have not registered a decision regarding organ donation, you will be treated as having no objection to being an organ donor.

If the **opt-out** system is introduced it means you will have **3 options:**

**Option 1.** Join the Organ Donor Register (ODR) if you want to be a donor (**opt-in**). 
**Option 2.** Record that you do not wish to be a donor (**opt-out**). 
**Option 3.** If you select neither option 1 or 2, you will be treated as having no objection to donating your organs (**deemed consent**).
