# Supplementary material for: ‘It’s like being conscripted, one volunteer is better than 10 pressed men’: A qualitative study into the views of people who plan to opt‐out of organ donation
Source: Br J Health Psychol. 2020 Jan 30;25(2):257–74. doi: 10.1111/bjhp.12406 (PMC7216962; doi:10.1111/bjhp.12406)
Supplement: Supplementary file 2 — Supporting information 2: Semi structured interview schedule. [file BJHP-25-257-s002.docx]

**Supporting information 2: Semi-structured interview schedule**

1. **(Opening question)** To begin, could you tell me more about your personal views on organ donation in general?

**Definition of the current opt-in donor system**: Currently in Scotland, England and Northern Ireland if you want to be an organ donor you must actively sign-up and join the organ donor register.

**2.** What do you think about the current opt-in donor system?

**3.** Do you think there are any particular good points to the current opt-in system?

**4.** Do you think there are any particular negative points to the current opt-in system?

**Definition of the proposed opt-out donor system**: The organ donation laws are planning to change. At the moment, in Scotland, England and Northern Ireland if you want to be an organ donor you must actively sign-up and join the donor register.

Recently, the Scottish and English Governments have announced plans to change organ donor laws to an opt-out system. This would remove the requirement to sign up and instead follows presumed consent. This means that if you take no action, you will become an organ donor by default. If you don’t want to be an organ donor you must actively remove yourself from the donor register, thereby opt-ing out.

**5.** What do you think about the plans to move to opt-out?

**6.** Can you describe any positives of an opt-out consent system?

**7.** Can you describe any negatives of an opt-out consent system?

**Orienting participants to time**: So, we’re just about finished so thanks for your patience. I’ve just got a few more questions left

**8.** Do you feel that you have any worries or fears about organ donation?

**9.** Do you think your view on organ donation would be different if the law changes to an opt-out system?

**10.** Do you have any suggestions for us as researchers on things that may make a difference? **11. (Closing question)** I think that’s everything I wanted to ask, is there anything else you’d like to say or any final thoughts you have?
